# Low Frequency Ventilation During Cardiopulmonary Bypass to Protect Postoperative Lung Function in Cardiac Valvular Surgery: The PROTECTION Phase II Randomized Trial

**DOI:** 10.1161/JAHA.124.035011

**Published:** 2024-09-30

**Authors:** Chris A. Rogers, Graziella Mazza, Rachel Maishman, Russell Thirard, Jonathan Evans, Samantha de Jesus, Chloe Beard, Gianni Angelini, Ann Millar, Nabil Jarad, Sally Tomkins, James Hillier, M‐Saadeh Suleiman, Raimondo Ascione

**Affiliations:** ^1^ Bristol Trials Centre, Bristol Medical School University of Bristol UK; ^2^ Faculty of Life and Health Sciences, Bristol Heart Institute University of Bristol UK; ^3^ Respiratory Medicine Southmead Hospital Bristol UK; ^4^ Department of Respiratory Medicine University Hospital Bristol and Weston NHS Foundation Trust Bristol UK

**Keywords:** cardiopulmonary bypass, low frequency ventilation, lung protection, pulmonary function tests, sRAGE, valvular surgery, Cardiovascular Surgery, Treatment

## Abstract

**Background:**

Cardiac surgery with cardiopulmonary bypass (CPB) triggers pulmonary injury. In this trial we assessed the feasibility, safety, and efficacy of low frequency ventilation (LFV) during CPB in patients undergoing valvular surgery.

**Methods and Results:**

Patients with severe mitral or aortic valve disease were randomized to either LFV or usual care. Primary outcomes included release of generic inflammatory and vascular biomarkers and the lung‐specific biomarker sRAGE (soluble receptor for advance glycation end products) up to 24 hours postsurgery. Secondary outcomes included pulmonary function tests and 6‐minute walking test up to 8 weeks postdischarge. Sixty‐three patients were randomized (33 LFV versus 30 usual care). Mean age was 66.8 years and 30% were female. LFV was associated with changes of sRAGE (soluble receptor for advance glycation end products) levels (geometric mean ratio, 3.05; [95% CI, 1.13–8.24] 10 minutes post CPB, and 1.07 [95% CI, 0.64–1.79], 0.84 [95% CI, 0.55–1.27], 0.67 [95% CI, 0.42–1.07], and 0.62 [95% CI, 0.45–0.85] at 2, 6, 12, and 24 hours post CPB respectively). No changes were observed for any of the generic biomarkers. Respiratory index soon after surgery (mean difference, −0.61 [95% CI, −1.24 to 0.015] 10 minutes post end of CPB), forced expiratory volume after 1 second/forced vital capacity ratio (0.050 [95% CI, 0.007–0.093] at 6 to 8 weeks pos‐surgery), Forced vital capacity alone (95% CI, −0.191 L [−0.394 to 0.012]) and 6‐minute walking test score at discharge (63.2 m [95% CI, 12.9–113.6]) were better preserved in the LFV group. No other differences were noted.

**Conclusions:**

The use of LFV during CPB in patients undergoing valvular surgery was feasible and safe and was associated with changes in sRAGE levels along with better preserved lung function and walking performance. These observations warrant further investigation in larger future studies.

**Registration:**

URL: https://www.isrctn.com; Unique Identifier: ISRCTN75795633.

**Nonstandard Abbreviations and Acronyms**
6MWT: 6‐minute walking test
CPB: cardiopulmonary bypass
LFV: low frequency ventilation
MV: mitral valve
sRAGE: soluble receptor for advance glycation end products
UC: usual care

**Clinical Perspective**

**What Is New?**

- This study suggests that the use of low frequency ventilation during cardiopulmonary bypass in the targeted valvular population is feasible and safe.
- In addition, it suggests that low frequency ventilation is associated with marked postoperative changes in circulating levels of the lung‐specific mediator sRage (soluble receptor for advance glycation end products), better preservation of lung function up to 8 weeks after surgery, and improved walking performance at hospital discharge.

**What Are the Clinical Implications?**

- A future large phase III clinical trial powered on strong clinical primary outcomes and confirming the clinical efficacy of the proposed method could trigger future adoption of low frequency ventilation in routine clinical practice. In addition, if future mechanistic studies identify sRAGE as a key mediator during cardiopulmonary bypass‐related lung ischemia–reperfusion injury, this might lead to the development of new drug‐based treatments.

Pulmonary injury during cardiac surgery with cardiopulmonary bypass (CPB) is still common.^1^, ^2^, ^3^, ^4^, ^5^ Predisposing factors include general anesthesia, median sternotomy, prolonged CPB,^1^, ^2^, ^3^, ^4^, ^5^ intraoperative lung collapse,^6^ inflammatory activation,^7^, ^8^, ^9^, ^10^ and perioperative blood transfusion.^11^, ^12^ To reduce postoperative lung injury, several methods of open‐lung ventilation during CPB have been tested over the years with conflicting results.^13^, ^14^, ^15^, ^16^, ^17^, ^18^, ^19^, ^20^ A previous phase II trial in a mixed group of 62 patients undergoing coronary and valvular surgery^13^ and preclinical studies by our group in large animals^14,15^ suggested that open‐lung methods during CPB might reduce inflammatory activation. However, a small phase II trial in patients undergoing only coronary artery bypass grafting (CABG) by our group suggested that the use of low frequency ventilation (LFV) during CPB did not reduce inflammatory activation and did not preserve perioperative lung function.^16^ Recent larger trials have tested several open‐lung methods during cardiac surgery in different mixed surgical populations showing no benefits in predefined outcome measures.^17^, ^18^, ^19^, ^20^, ^21^ However, there is little in the literature on testing open‐lung treatments during CPB in selected patients with severe mitral or aortic valve disease undergoing open cardiac valvular surgery. Of note, previous studies assessing new open‐lung treatments during cardiac surgery in small phase I and II trials have focused on generic biomarkers rather than lung‐specific biomarkers.^13^, ^14^, ^15^, ^16^, ^17^, ^18^, ^19^, ^20^, ^21^ The sRAGE (soluble receptor for advance glycation end) product is a well‐established biomarker of lung‐specific injury that is used regularly in respiratory medicine, with changes in sRAGE circulating levels being associated with a variety of pulmonary conditions.^22^, ^23^, ^24^, ^25^, ^26^, ^27^ The aim of this phase II randomized controlled trial was to assess the feasibility, safety, and functional efficacy of LFV during CPB in selected patients with severe mitral or aortic valve disease undergoing open cardiac valvular surgery.

## Methods

### Study Design and Data Availability

A single‐center parallel‐group randomized controlled phase II trial. Participants were allocated in a 1:1 ratio to receive LFV during CPB or usual care (UC). Study participants were followed up for 6 to 8 weeks after discharge. The trial was registered (ISRCTN75795633). The full study protocol is available at the Bristol Trials Centre, Bristol, UK, a UKCRC registered clinical trials unit.

The data underlying this article are available in this article and its supplementary online files. The raw data underlying the reported findings will be shared on reasonable request to the corresponding author.

### Target Population

Elective or urgent patients with severe mitral valve (MV) or aortic valve disease, 18 years of age or older, who had been referred for valve repair or replacement surgery were selected for this study. In all cases, surgery was to be undertaken under general anesthesia via median sternotomy, CPB, and aortic cross‐clamping. Patients with left ventricular ejection fraction >25%, severe MV, or aortic valve stenosis or regurgitation were included. To minimize bias and confounding exclusion criteria included CABG alone, redo surgery, aortic surgery, pulmonary embolism requiring anticoagulation >3 months, heart failure (New York Heart Association class IV), cardiogenic shock, chronic renal failure requiring dialysis, corticosteroid or immunosuppressive treatments, emergency/salvage operation, sepsis, or acute endocarditis. The diagnosis of severe MV or aortic valve stenosis or regurgitation was made by independent senior cardiologists based on standardized protocols and established echocardiographic guidelines. The decision to go for open surgical treatment was made via the Heart Team. The decision to exclude patients who had CABG alone was made by the Trial Steering Committee before starting patient recruitment based on the results of a small pilot study indicating no benefit of LVF in patients who had CABG alone.

### Study Settings

The study was conducted at the Bristol Heart Institute, a regional cardiac surgery center in the United Kingdom, according to the principles outlined in the Declaration of Helsinki. The University Hospital Bristol and Weston NHS Foundation Trust sponsored the trial. The Southmead Research Ethics Committee gave ethical approval including subsequent design amendments (Ref. CS/2009/3259) and all recruited patients gave informed consent. The trial, data collection, and data analysis were managed independently by the registered Bristol Clinical Trial Evaluation Unit (now Bristol Trials Centre—BTC, Bristol, UK).

### Anesthesia, Mechanical Ventilation, and Open‐Lung Interventions During CPB

Consented patients were randomized to receive either LFV or usual care during CPB. Anesthesia, mechanical ventilation, CPB, surgical methods, and postoperative management were based on standardized protocols^28^ with more details provided in Data S1. Briefly, following a premedication, anesthesia was induced with a combination of midazolam, propofol, and fentanyl 5‐10 mcg/kg, and muscle relaxation was achieved with vecuronium or rocuronium. Anesthesia was maintained with a combination of volatile anesthetic agent, propofol (2–6 mg/kg h) and fentanyl (5 mcg/kg up to maximum of 20 mcg/kg). Mechanical ventilation was the same in both groups and guided by routine clinical practice entailing a positive end‐expiratory pressure (PEEP) of 5 cmH_2_O and normal volume control ventilation at the following settings: tidal volume of 6 to 8 mL/kg, inspiratory/expiratory ratio of 1:2, FiO_2_ of 100% for 5 minutes to be reduced to 50% thereafter, and ventilation rate of 12/min aiming to obtain a favorable PaCO_2_ (35.0–45.0 mm Hg) and pH (7.35–7.45).

#### Low Frequency Ventilation

Once patients were on CPB, the ventilation was set up at a respiratory rate of 5 inflations/min with air (21% oxygen) at a tidal volume of 6 to 8 mL/kg and PEEP of 5 cmH_2_O according to a standardized protocol. Just before weaning CPB a standardized lung recruitment maneuver was performed by increasing peak inspiratory pressure to 40 cmH_2_O for 15 seconds. Next, the ventilation was kept at strict pre‐CPB settings until the patients met the criteria for extubation.

#### Usual Care

Once patients were on CPB, the lungs were disconnected from the ventilator and left collapsed for the entire CPB duration. Just before CPB weaning, the same lung recruitment maneuver described in the LVF group was performed by increasing peak inspiratory pressure to 40 cmH_2_O for 15 seconds. Then, the ventilation was kept at routine pre‐CPB settings, until the patients met the criteria for extubation.

### Outcome Measures

#### Primary Biochemical Outcomes

The primary outcomes included the following generic biomarkers: TNFα (tumor necrosis factor alpha); IL1β, IL‐6, IL‐10 (interleukin‐1beta, 6, and 10), TXA2 (thromboxane A2); sICAM‐1 (soluble intercellular adhesion molecule‐1); sVCAM‐1 (soluble vascular adhesion molecule‐1); tPAI‐1 (tissue plasminogen activator inhibitor‐1); sphingosine 1‐phosphate^29^, ^30^, ^31^; and 1 lung‐specific biomarker (sRAGE) used regularly in respiratory medicine.^22^, ^23^, ^24^, ^25^, ^26^, ^27^ All biomarkers were measured serially in blood samples taken at baseline (postinduction of anesthesia), 10 minutes and 2, 6, 12, and 24 hours after CPB weaning.

#### Secondary Functional Outcomes

These included sensitive pulmonary function tests routinely used in clinical practice:

1. *Pulmonary function tests*: Forced vital capacity (FVC), forced expiratory volume after 1 second (FEV1) and FEV1/FVC ratio measured before surgery, at predischarge, and at 6 to 8 weeks postsurgery.
2. *Pulmonary oxygen exchange*: Alveolar‐arterial oxygenation gradients (PAO_2_–PaO_2_) and respiratory index [(PAO_2_–PaO_2_)/(PaO_2_)] measured at postinduction, 10 minutes, 2, 4, and 12 hours after CPB weaning derived from serial hemogas analysis. Increase of alveolar‐arterial oxygenation gradient and of respiratory index indicate greater hypoxemia due to impaired oxygen exchange.
3. *Lung static and dynamic compliance*: Peak airway pressure (cmH_2_O) measured at 2 and 4 hours after CPB weaning.
4. *Trapping of white blood cells*: Left atrial/right atrial (LA/RA) white blood cell ratio measured before institution of CPB and at CPB weaning. A left atrial/right atrial white blood cell ratio <1 indicates trapping of leukocytes and platelets in the lungs.
5. *Functional exercise capacity*: All patients were subjected to a 6‐minute walking test (6MWT) at predischarge. The 6MWT is a commonly used test for the objective assessment of functional exercise capacity in patients with moderate‐to‐severe pulmonary disease.^32^, ^33^

### Clinical Outcome

Generic clinical outcome included bacteriologically proven chest infection, mechanical ventilation >24 hours, postoperative need for continuous positive airway pressure, reintubation, tracheostomy, or acute respiratory distress syndrome up to 8 weeks after surgery. In addition, a generic time until fit for discharge was calculated based on the time point when the patients had achieved predefined targets including being afebrile, heart and respiratory rates within normal range, saturation ≥96% on room air, normal bowel function, and being physically mobile.

### Randomization and Blinding

Randomization sequence was according to a computer‐generated list drawn up by an independent operator before the start of the trial. Cohort minimization was used to achieve balanced distribution between groups of surgical procedure and poor baseline lung function (FEV1 <60% predicted). Allocations were concealed until a participant was recruited and registered onto a secure purpose‐designed electronic database. Randomization took place the evening before surgery; if a participant's surgery was unexpectedly rescheduled, they retained their randomized allocation. Data were collected anonymously on the electronic platform and each patient was assigned a unique identification number. Participants, hospital staff not involved in the surgery, and research personnel responsible for data collection and laboratory analyses were blinded to the treatment allocation.

### Statistical Analysis

All biomarkers were measured longitudinally over 6 consecutive time points to allow detection of a large effect in a small phase II trial.^8^, ^26^ A sample size of 60 participants was chosen to provide 90% power to detect a difference in biomarkers of 0.60 SDs at the 5% significance (2 tailed), assuming a correlation of 0.5 between pre and postintervention measures and a correlation of 0.7 between repeated postintervention measures. All biomarkers were measured in duplicates, both included in the statistical analysis. Analyses were performed only for predefined primary biochemical and secondary functional outcomes on an intention‐to‐treat basis in line with a prespecified analysis plan. Outcomes were compared using regression methods. Models included treatment allocation, stratification variables, and baseline measures (where available) fitted as fixed effects. Longitudinal outcome models also included time (category variable) and treatment by time as fixed effects and participant and time fitted as random effects. If the interaction between treatment group and time was statistically significant at *P*<0.1, results are presented for each time point separately, otherwise the treatment by time interaction was removed from the model and an overall treatment effect is given. Alternative variance/covariance structures were compared (using likelihood ratio tests) to best allow for the correlation between measurements taken at different times for the same participant. Likelihood ratio tests were used to determine statistical significance, and 2‐tailed *P* values <0.05 were considered statistically significant. Results are reported as effect sizes with 95% CIs. Analyses were performed in Stata version 16.1 (Stata Corp LP, College Station, TX) or SAS version 9.4 (SAS Institute Inc, Cary, NC). Additional information on the statistical analysis is provided in Data S1.

## Results

**Figure 1:**
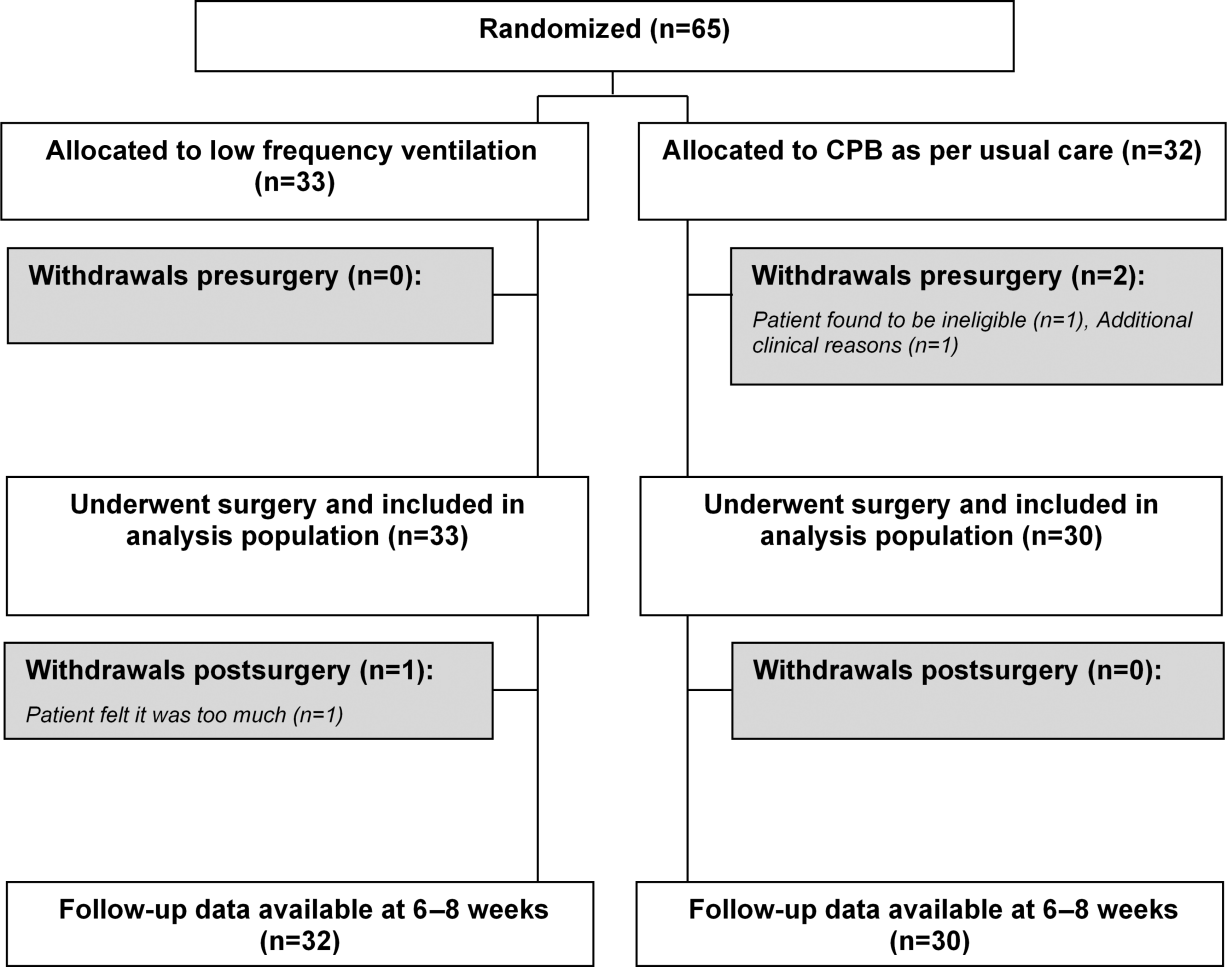
CONSORT flow chart. CONSORT flow chart illustrating the allocation of patients between groups, exclusions, withdrawals overtime and follow‐up. CONSORT indicates Consolidated Standards of Reporting Trials; and CPB, cardiopulmonary bypass.

This phase II trial is reported according to the Consolidated Standards of Reporting Trials guidelines. The flow chart is shown in Figure 1.

### Patient Recruitment and Timing of Data Collection/Validation, Laboratory Assays, and Data Analysis

Between January 5, 2011 and July 16, 2012, 65 eligible patients were recruited and randomized, 33 to LFV and 32 to UC. Participants were followed up 6 to 8 weeks after surgery.

Two participants randomized to UC were withdrawn at surgery in line with study protocol due to intraoperative findings indicating additional procedures needed. One participant in the LVF group withdrew after discharge (Table S1). All patients in the treatment group received LVF according to the predefined protocol. On completion of patient recruitment, clinical data collection and subsequent validation and database locking were completed by 2015. All biomarker laboratory assays were undertaken in duplicate with data validation completed by late 2015. All molecular and functional data were analyzed from mid‐2018 to mid‐2019 due to a gap in funding. Article preparation and its final approval by the Trial Unit lead was markedly delayed by the pandemic outbreak.

### Baseline Characteristics and Operative Data

Baseline characteristics, type of valvular disease, operative details, and preoperative medications are shown in Tables 1 and 2 and Tables S2 and S3. The mean age was 66.8 years (SD 10.7), with 19/63 (30.2%) of patients being female. Of the 63 patients 28 were in New York Heart Association Class III and 12 had left ventricular ejection fraction ≤50%. All 63 patients were diagnosed with severe aortic or mitral valve stenosis or regurgitation based on baseline echocardiographic findings. Severe aortic or mitral valve stenosis or severe regurgitation were equally distributed among groups (Table 1).

**Table 1:** Baseline Characteristics

	Randomized to LFV (n=33)		Randomized to usual care (n=30)		Overall (n=63)	
	n	%	n	%	n	%
Baseline characteristics						
Age, y (SD)	64.9	10.0	68.9	11.1	66.8	10.7
Female sex	6/33	18.2%	13/30	43.3%	19/63	30.2%
Body mass index, kg/m^2^						
Mean±SD	28	5.3	26	4.2	27	4.9
New York Heart Association class						
I–II	19/33	57.6%	16/30	53.3%	35/63	55.5%
III–IV	14/33	42.4%	14/30	46.7%	28/63	44.5%
Canadian Cardiovascular Society class						
Asymptomatic	24/33	72.7%	23/30	76.7%	47/63	74.6%
I–II	7/33	21.2%	5/30	16.7%	12/63	18.0%
III–IV	2/33	6.1%	2/30	6.6%	4/63	6.4%
Echocardiographic and angiographic results						
Left ventricle function						
Good (>50%)	28/33	84.8%	23/30	76.7%	51/63	81.0%
Moderate (30%–50%)	4/33	12.1%	6/30	20.0%	10/63	15.9%
Poor (<30%)	1/33	3.0%	1/30	3.3%	2/63	3.2%
Valve disease alone	30/33	90.9%	24/30	80.0%	54/63	85.7%
Type of valve disease						
Aortic (S/R)	9 (7/2)	27.3%	15 (11/4)	50.0%	24 (18/6)	38.1%
Mitral (S/R)	25 (8/17)	75.8%	16 (5/11)	53.3%	41 (13/28)	61.9%
Valve and CAD disease	3/33	9.1%	6/30	20.0%	9/63	14.3%
Extent of CAD						
Single	0/33	0.0%	4/30	13.3%	4/63	6.3%
Double	2/33	6.1%	0/30	0.0%	2/63	3.2%
Triple	1/33	3.0%	2/30	6.7%	3/63	4.8%
Past medical history						
Smoking status						
Smoker	2/33	6.1%	4/30	13.3%	6/63	9.5%
Exsmoker	10/33	30.3%	13/30	43.3%	23/63	36.5%
Nonsmoker	21/33	63.6%	13/30	43.3%	34/63	54.0%
Asthma	2/33	6.1%	1/30	3.3%	3/63	4.8%
Chronic obstructive pulmonary disease	0/33	0.0%	1/30	3.3%	1/63	1.6%
Family history of lung disease	7/33	21.2%	4/30	13.3%	11/63	17.5%
Nonlung disease						
Treated hypertension	16/33	48.5%	16/30	53.3%	32/63	50.8%
Hypercholesterolemia	11/33	33.3%	11/30	36.7%	22/63	34.9%
Hypothyroidism	3/33	9.1%	1/30	3.3%	4/63	6.3%
Diabetes						
Insulin	1/33	3.0%	0/30	0.0%	1/63	1.6%
Oral	3/33	9.1%	2/30	6.7%	5/63	7.9%
Diet	2/33	6.1%	0/30	0.0%	2/63	3.2%
Cerebral vascular accident/transient ischemic attack	2/33	6.1%	1/30	3.3%	3/63	4.8%
EuroSCORE						
Mean±SD	2	1.8	4	1.5	3	1.8
Arrhythmias						
Atrial fibrillation	12/33	36.4%	10/30	33.3%	22/63	34.9%
Block	0/33	0.0%	1/30	3.3%	1/63	1.6%
Peptic ulceration	1/33	3.0%	0/30	0.0%	1/63	1.6%
Operative priority						
Elective	32/33	97.0%	27/30	90.0%	59/63	93.7%
Urgent	1/33	3.0%	3/30	10.0%	4/63	6.3%

Missing data: body mass index: data missing for 1 patient (0, 1); EuroSCORE: data missing for 2 patients (0, 2). CAD indicates coronary artery disease; LFV, low frequency ventilation; and S/R, stenosis/regurgitation.

**Table 2:** Operative Details

	Randomized to LFV (n=33)		Randomized to usual care (n=30)		Overall (n=63)	
	n	%	n	%	n	%
Bypass data						
Operation time (min)						
Median (IQR)	280	(245.0–305.0)	240	(215.0–300.0)	265	(225.0–305.0)
Cardiopulmonary bypass time (min)						
Median (IQR)	115	(102.0–146.0)	112	(94.0–129.0)	112	(98.0–131.0)
Cardioplegic arrest time (min)						
Median (IQR)	84	(72.0–101.0)	79	(67.0–92.0)	81	(67.0–100.0)
Lowest hematocrit						
Median (IQR)	28	(23.2–30.5)	26	(23.2–28.0)	27	(23.2–29.3)
Lowest core temperature						
Median (IQR)	32	(31.8–32.0)	32	(31.9–32.0)	32	(31.9–32.0)
Blood loss*						
Median (IQR)	400	(300.0–850.0)	525	(375.0–650.0)	475	(325.0–700.0)
Blood saving techniques						
Tranexamic acid	33/33	100.0%	29/30	96.7%	62/63	98.4%
Use of cell saver	5/33	15.2%	4/30	13.3%	9/63	14.3%
Red blood cell transfusion	9/33	27.3%	6/30	20.0%	48/63	23.8%
Units transfused						
Median (IQR)	1.0	(1.0–2.0)	1.5	(1.0–3.0)	1	(1.0–2.0)
Fresh frozen plasma transfusion	4/33	12.1%	2/30	6.7%	6/63	9.5%
Median (IQR)	3.5	(2.5–4.0)	1.5	(1.0–2.0)	3	(2.0–4.0)
Platelet transfusion	5/33	15.2%	5/30	16.7%	10/63	15.9%
Median (IQR)	2	(1.0–2.0)	1	(1.0–1.0)	1	(1.0–2.0)
Activated factor VII	1/33	3.0%	0/30	0.0%	1/63	1.6%
Intraoperative arrythmias						
Arrhythmias on removal of cross clamp						
Atrioventricular block	7/31	22.6%	6/30	20.0%	13/61	21.3%
Atrial fibrillation/flutter	6/31	19.4%	8/30	26.7%	14/61	23.0%
Ventricular tachyarrhythmia/ventricular fibrillation	6/31	19.4%	2/30	6.6%	3/61	4.9%
Temporary pacing						
Single chamber	11/33	33.3%	10/30	33.3%	21/63	33.3%
Dual chamber	13/33	39.4%	10/30	33.3%	23/63	36.5%
Other intraoperative details						
Need for insulin infusion	10/33	30.3%	3/30	10.0%	13/63	20.6%
Need for mild inotropes	9/33	27.3%	4/30	13.3%	13/63	20.6%
Need for noradrenaline	12/33	36.4%	5/30	16.7%	17/63	27.0%
Need for vasodilator	2/33	6.1%	1/30	3.3%	3/63	4.8%
Type of valve surgery						
Aortic valve (n=24)						
Repaired	0/33	0.0%	0/30	0.0%	0/63	0.0%
Replaced	9/33	27.3%	15/30	50.0%	24/63	38.1%
Mitral valve (n=41)						
Repaired	9/33	27.3%	4/30	13.3%	13/63	20.6%
Replaced	16/33	48.5%	12/30	40.0%	28/63	44.4%

IQR indicates interquartile range; and LFV, low frequency ventilation.

* At first 12 h after surgery.

Overall, 41/63 patients had MV surgery and 24/63 had aortic valve surgery, with similar distribution of repair/replacement rates between both groups. Only 9/63 patients required some bypass grafting on top of their valvular surgery due to concomitant severe coronary disease. of the 63 patients, 29 were current or exsmokers with similar distribution between groups (Table 2). Baseline distribution of all medications, including those needed to treat chronic lung disease are shown in Table S2. In addition, baseline FVC, FEV1, and FEV1/FVC ratio did not differ between groups (Table 3). There were trends for mean EuroSCORE to be slightly higher in the LFV group, whereas diabetes and MV disease were slightly higher in the LFV group. CPB time did not differ between groups with mean LFV duration being 115 minutes. Duration of surgery was 40 minutes longer in the LFV group (Table 2).

**Table 3:** Secondary Functional Outcome: Serial Pulmonary Function Tests

	Randomized to LFV (n=33)		Randomized to usual care (n=30)		Effect^1^ MD (95% CI)*	*P* value
	Mean	SD	Mean	SD		
FVC						
Before surgery	3.52	0.90	3.24	1.19	…	…
Before discharge	2.16	0.47	2.24	0.77	…	…
6–8 wk post discharge	3.29	0.86	3.26	1.03	…	…
Treatment×Time interaction	…	…	…	…	…	0.95
Overall	…	…	…	…	−0.191 (−0.394 to 0.012)	0.05
FEV1						
Before surgery	2.60	0.69	2.30	0.79	…	…
Before discharge**	1.60	0.39	1.60	0.55	…	…
6–8 wk post discharge^†^	2.47	0.67	2.22	0.76	…	…
Treatment×Time interaction	…	…	…	…	…	0.33
Overall	…	…	…	…	−0.135 (−0.290 to 0.019)	0.08
FEV1/FVC						
Before surgery	0.74	0.08	0.72	0.11	…	…
Before discharge*	0.74	0.09	0.73	0.13	−0.0005 (−0.044 to 0.043)	0.98
6–8 wk post discharge^†^	0.75	0.10	0.68	0.10	0.050 (0.007 to 0.093)	0.02
Treatment×time interaction	…	…	…	…	…	0.04
Alveolar‐arterial oxygenation gradient						
Preoperative^‡^	6.59	86.02	17.92	16.08	…	…
Postinduction^§^	146.46	76.10	128.08	93.33	…	…
10 min post end of CPB^||^	125.13	81.89	164.44	111.40	…	…
Before chest closure^#^	150.40	67.84	157.62	89.62	…	…
2 h post CPB^§^	160.27	70.64	150.22	96.96	…	…
4 h post CPB**	159.24	74.31	146.17	97.14	…	…
12 h post CPB^††^	238.70	182.46	229.77	190.50	…	…
Treatment×time interaction	…	…	…	…	…	0.45
Overall	…	…	…	…	6.755 (−25.192 to 38.702)	0.65
Respiratory index						
Preoperative^‡^	0.27	0.28	0.25	0.19	…	…
Postinduction^§^	1.01	0.98	0.74	0.76	0.252 (−0.193 to 0.697)	0.25
10 min post end of CPB^||^	0.77	0.84	1.40	1.50	−0.610 (−1.236 to 0.0147)	0.05
Before chest closure^#^	1.11	0.88	1.27	1.19	−0.132 (−0.673 to 0.409)	0.62
2 h post CPB^§^	1.49	1.23	1.17	0.95	0.273 (−0.272 to 0.818)	0.31
4 h post CPB**	1.17	0.80	1.03	0.75	0.136 (−0.253 to 0.524)	0.48
12 h post CPB^††^	1.90	1.67	2.20	2.81	0.023 (−0.818 to 0.863)	0.95
Treatment×time interaction	…	…	…	…	…	0.05
Peak airway pressure						
Presternotomy^‡‡^	18.53	4.64	18.24	3.72	…	…
2 h post CPB^§§^	16.62	3.38	17.64	3.73	…	…
4 h post CPB^||||^	17.54	3.38	18.67	3.99	…	…
Treatment×time interaction	…	…	…	…	…	0.91
Overall	…	…	…	…	−0.813 (−2.3448 to 0.718)	0.28
Left atrium/right atrium white blood cell count						
Before institution of CPB^##^	0.91	0.15	0.98	0.13	…	…
Weaning from CPB^§^	0.94	0.09	0.96	0.10	…	…
Overall	…	…	…	…	−0.027 (−0.074 to 0.021)	0.27

Treatment effect estimates are given either for each time point or overall, depending on whether a treatment×time interaction term is found to be required in the model. Missing data (LFV, usual care): CPB indicates cardiopulmonary bypass; FEV_1_, forced expiratory volume after 1 s; FVC, forced vital capacity; LFV, low frequency ventilation; and MD, mean difference.

*8(6, 2) ^†^8(5, 3) ^‡^1(0, 1) ^§^2(1, 1) ^||^4(3, 1) ^#^5(3, 2) **2(0, 2) ^††^4(2, 2) ^‡‡^9(3, 6) ^§§^10 (4, 6) ^||||^12(5, 7) ^##^3(1, 2).

Postoperatively, 7/63 patients required chest reopening (5/33 in LFV versus 2/30 in UC group). Nine patients in the LFV and 6 in the UC groups required intraoperative blood transfusion. Also, 11 participants in the LFV group and 15 in the UC group received blood transfusion (Table S3). Postoperative intubation time was 1 hour shorter in the LFV group (6.75 [4.25–9.75] versus 7.50 [4.25–12.63]; Table S3).

#### Primary Biochemical Outcomes

**Figure 2:**
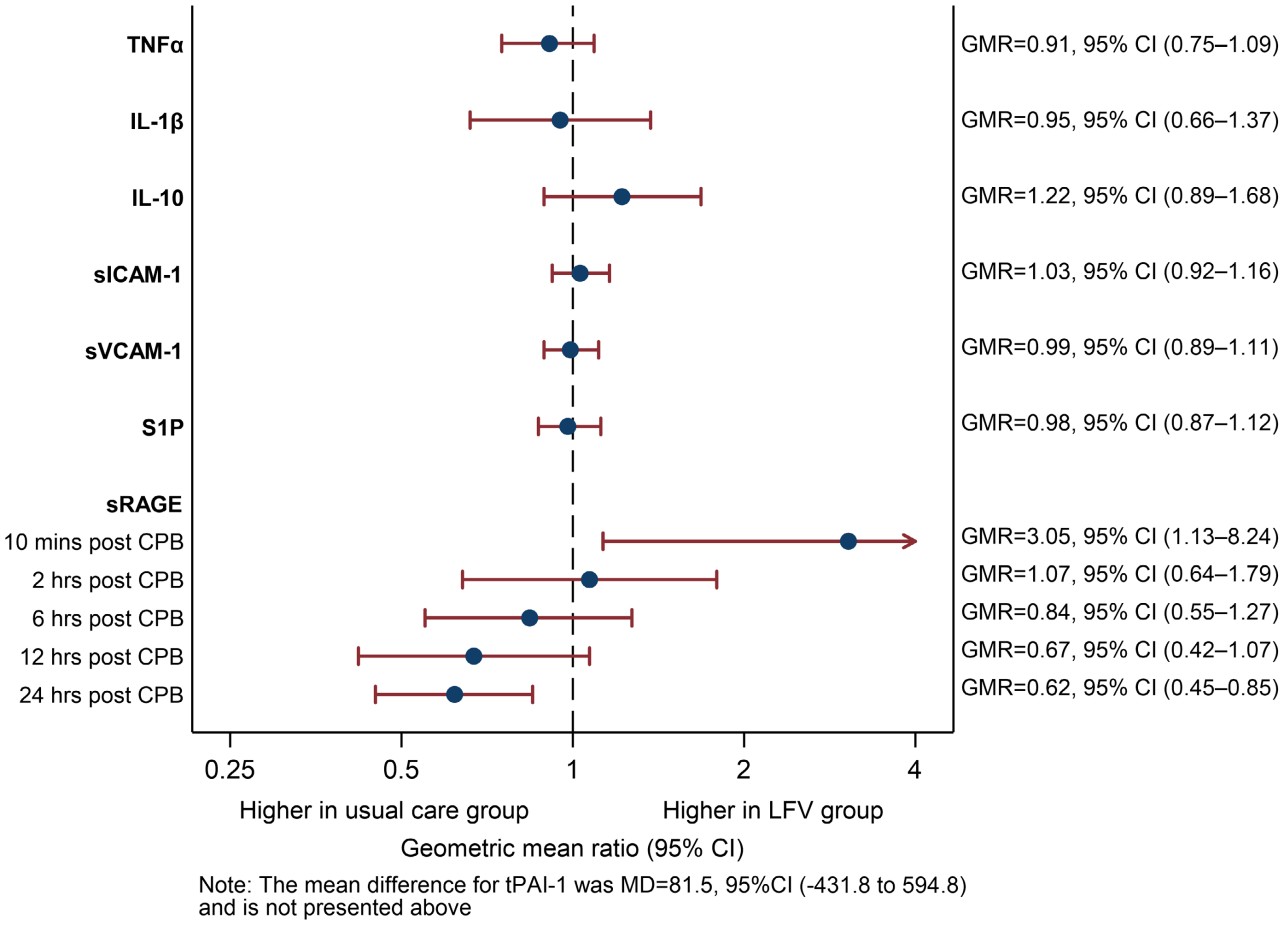
Effect of LFV on primary biomarkers outcome. Forest plot illustrating the treatment effect for each biomarker. All the biomarkers shown, with the exception of S1P, were reported in pg/mL. The mean difference for tPAI‐1 was MD=81.5, 95% CI (−431.8 to 594.8) and is not presented in the figure. S1P was reported in μg/L. CPB indicates cardiopulmonary bypass; GMR, geometric mean ratio; IL, interleukin; LFV, low frequency ventilation; MD, mean difference; TNFα, tumor necrosis factor alpha; tPAI‐1, plasminogen activator inhibitor‐1; S1P, sphingosine‐1‐phosphate; sICAM‐1, soluble intercellular adhesion molecules‐1; sRAGE, soluble receptor for advance glycation end products; and sVCAM‐1, soluble vascular cell adhesion molecules‐1.

Primary biochemical outcomes are shown in Figure 2, Table S4, and Figures S1 and S2.

None of the generic biomarkers tested showed a difference overtime between groups (Figure 2, Figures S1 and S2 and Table S4). With regard to sRAGE, the only lung‐specific biomarker used, the circulating levels were 3 times higher in the LFV group compared with the UC group at 10 minutes post CPB weaning (geometric mean ratio, 3.05 [95% CI, 1.13–8.24], *P*=0.026). Thereafter, the difference between groups gradually changed over to become significantly higher in the UC group at 24 hours post CPB weaning (geometric mean ratio, 0.62 [95% CI, 0.45–0.85], *P*=0.003) (Figure 2, Figure S2, and Table S4). TXA2 and IL‐6 were not detectable despite following strictly the manufacturer's instructions, hence they are not shown. All the other biomarkers measured are reported in pg/mL, with the exception of sphingosine 1‐phosphate, which is reported in μg/L.

#### Secondary Functional Outcomes

Secondary functional outcomes are shown in Table 3 and Figure S3. At baseline, FVC, FEV1, and FEV1/FVC ratio were balanced between groups. Postoperatively, the FVC was 0.19 L higher (95% CI, −0.012 to 0.394; *P*=0.05) and the FEV1 was 0.135 L higher (95% CI, −0.019 to 0.290; *P*=0.08) in the LFV group versus UC group. Before hospital discharge the FEV1/FVC ratio was similar in the 2 groups, but this was 5% (95% CI, 0.7% to 9.3%) higher in the LFV group at 6 to 8 weeks post discharge (*P*=0.02). Alveolar‐arterial oxygenation rose similarly from baseline in both groups (mean difference, 6.76 [95% CI, −25.2 to 38.7]) with no differences between groups. The respiratory index was better in the LFV group 10 minutes after CPB weaning (mean difference, −0.61 [95% CI, −1.24 to 0.0015], *P*=0.05) and overall (*P*=0.05). Peak airway pressure and left atrial/right atrial white blood cell ratio were similar between groups (Table 3). The 6MWT score at predischarge showed that the patients in the LFV group walked on average 63.2 m more (95% CI, 12.9 m–113.6 m) than those recruited in the UC group (*P*=0.012) (Table S5).

### Clinical and Safety Outcome

Postoperative clinical complications are reported only descriptively as the trial was not powered for these measures. These occurred overall in 9/63 patients, of whom 5/33 were in the LFV group and 4/30 in the UC group (relative risk, 1.24 [95% CI, 0.39–3.91]). Lung complications, time fit for discharge, and adverse events postsurgery up to 6 to 8 weeks post discharge are reported in Tables S5 through S7. There were no postoperative deaths, myocardial infarctions, permanent strokes, or cases of renal failure requiring dialysis. No differences were observed for serious adverse events, intensive care length of stay, or hospital length of stay.

## Discussion

This phase II trial suggests that the use of LFV during CPB is feasible and safe in patients undergoing MV or aortic valve surgery. In addition, it suggests that the use of LFV is associated with marked postoperative changes of sRAGE levels along with better preservation of few pulmonary function tests (respiratory index at 10 minutes after CPB weaning and overall, FVC and FEV1/FVC ratio at 6–8 weeks post discharge) and longer 6 minutes walking performance at predischarge.

The use of sRAGE was approved by the Trial Steering Committee on completion of patient recruitment and of the pilot laboratory validation at bench assay, which strictly followed manufacturer's instructions for all biomarkers. The rationale for measuring sRAGE was to have a lung‐specific biomarker of lung injury/preservation, already well known in respiratory medicine,^22^, ^23^, ^24^, ^25^, ^26^, ^27^ over and above the generic biomarkers. Although it is difficult at this stage to speculate if and how the marked changes in sRAGE levels might have influenced the observed changes in pulmonary function tests of this study due to lack of mechanistic data, in respiratory medicine it has been suggested that sRAGE may act as mediator of protective responses to cell injury triggered by oxidative stress and hypoxia^23^ by binding advanced glycation end‐products in the extracellular fluid, hence preventing their binding with RAGE.^27^, ^34^ High circulating levels of sRAGE have been associated with acute lung conditions such as acute respiratory distress syndrome and trauma,^22^, ^23^, ^24^ as well as with lung injury triggered by high tidal volumes during mechanical ventilation.^22^ At the same time, low levels of sRAGE have been linked with nonacute lung disease such as idiopathic pulmonary fibrosis,^25^ acquired lung fibrosis,^26^ and chronic obstructive lung disease,^27^ with speculations that higher sRAGE levels in these patients might be beneficial.

The PROVECS (Protective Ventilation in Cardiac Surgery) trial tested, in a mixed group of 500 patients undergoing cardiac surgery, a complex open‐lung method involving high PEEP (8 cmH_2_O) and lung recruitment maneuvers with continuous positive airway pressure at 30 cmH_2_O for 30 seconds repeated 5 times during surgery and up to extubation time; the control group involved only low PEEP (2 cmH_2_O), no ventilation during CPB, and no recruitment maneuvers.^17^ The PROVECS trial showed no differences of primary composite of postoperative pulmonary complications. In addition, 2 later subanalyses of this trial in 56 and 30 patients showed no effects on postoperative lung function and sRAGE levels respectively, the latter representing the only cardiac surgery study reporting perioperative sRAGE levels so far.^18^ The major differences in open‐lung methods and targeted patient population occurred between PROVECS and the present study might explain the different results observed with regard to sRAGE release and pulmonary function tests, but this speculation requires further validation.

Pulmonary function tests and 6MWT are used extensively in respiratory medicine for diagnostic or therapeutic decision‐making.^22^, ^23^, ^24^, ^25^, ^26^, ^27^, ^33^, ^34^, ^35^ They are also used in cardiac surgery for preoperative risk profiling of patients to support informed consenting, as their very high sensitivity helps establish levels of preoperative lung injury/dysfunction, ascertaining fitness for surgery and guiding postoperative care. In this study, serial pulmonary tests and 6MWT were undertaken by staff blinded to the allocation. Lung function tests were used first to assess the severity of preoperative pulmonary dysfunction with a view to balancing the distribution of those cases with marked lung dysfunction among groups to avoid confounding. In addition, they were used postoperatively to assess the safety of LVF during valvular surgery as well as the possible benefits of LFV on pulmonary function. The findings of these well‐established pulmonary tests not only confirm the safety of the proposed LFV method but also suggest that LFV may be associated with better postoperative preservation of the respiratory index, FEV_1_/FVC ratio, and FVC alone, along also with a trend for better FEV_1_ alone (Table 3). These results appear to be in keeping with a better 6MWT performance in the LVF group at predischarge.

It might be argued that the findings of this study may conflict with those of other studies assessing the safety/efficacy of open‐lung methods in patients undergoing cardiac surgery. However, such a comparison may not be meaningful as there are marked differences in open‐lung methods, targeted patient populations and outcome measures used across these different studies. For example, the PROVECS trial^17^, ^18^ not only tested a more complex open‐lung method but also recruited a very mixed population (28% of CABG only, 25% of thoracic aorta or combined procedures, and 47% of valve only patients) with no balancing for severe preoperative lung dysfunction across groups. In the present phase II trial the LFV method used was simple and applied only during CPB (all other aspects of care were similar between groups), whereas among all patients who underwent valvular surgery 9/63 cases received bypass grafts on top of valvular surgery, which were equally distributed between groups (Table 1). Another trial in 1500 patients who received cardiac surgery tested a simple open‐lung method only during CPB (5 breath/min; 3 mL/kg of tidal volume and PEEP of 5 cmH_2_O), reporting no difference between groups.^19^ Again, the targeted population was quite mixed (>50% of CABG alone cases along with acute endocarditis and redo cardiac surgery cases). Surprisingly, 172/756 patients recruited in the open‐lung group did not receive the intended treatment and this represented a major methodological weakness. A further trial recruited 413 patients who received cardiac surgery across 3 groups (no open‐lung method, open‐lung at low tidal volume, or open‐lung at high tidal volume during CPB), reporting better oxygenation index in the open‐lung groups versus control, but no effect on the primary composite of postoperative complications.^20^ Finally, a meta‐analysis pooled 15 small trials (738 patients in total) despite the trials using very different open‐lung methods and 13/15 of the trials recruiting patients undergoing CABG alone.^21^ The study reported no benefits associated with the pooled open‐lung methods.

This study has limitations. Despite randomization and blinding, minor heterogeneities occurred by chance in distribution of some baseline factors, with trends toward less MV disease and diabetes in the usual care group and a slightly lower EuroSCORE in the LFV group. In addition, the outcome measures focused on biochemical and pulmonary function tests given their high sensitivity as this was a phase II small trial. We appreciate that focusing on rare and more robust clinical outcomes would have required a much larger phase III trial. It might be argued that LFV is not justified as this trial did not show differences for the other generic biomarkers and clinical outcomes tested. However, expecting LFV to affect generic biomarkers such as inflammatory markers may not be realistic given that in both groups patients required prolonged CPB and cardioplegic arrest times that are well‐known determinants of postoperative inflammatory activation. Equally, expecting benefits in clinical outcome triggered by LFV may also not be realistic as this was a phase II trial not powered for clinical end points. Also, it could be argued that a subanalysis might have clarified whether the impact of LVF might have been different on patients with aortic valve disease versus those with mitral valve disease given their possible different effects on pulmonary circulation. However, we did not undertake this subanalysis due to it not being prespecified in our analysis plan and the subgroups being too small to provide meaningful additional information. Finally, there was delay from completion of patient recruitment and follow‐up to submission of this paper for publication. However, the quality of our findings was not affected as rigorous methodological approaches were used throughout and for each methodological aspect of the study. Finally, the anesthetic and surgical techniques used during the patient recruitment period are similar to those used currently, making the findings of this study still relevant to current practice.

### Conclusions

In conclusion, in this phase II trial the use of LFV during CPB in patients undergoing valvular surgery was feasible and safe. In addition, LFV triggered postoperative changes in sRAGE levels along with better preservation of few lung function tests and 6MWT performance in the postoperative period. Although these observations are interesting, they warrant further investigation in future larger studies with focus on clinical end points.

## Sources of Funding

The trial was led by Raimondo Ascione, who also performed most of the surgery. The trial was supported by the National Institute of Health Research Bristol Cardiovascular Biomedical Research Unit. Raimondo Ascione is supported by grants from the National Institute of Health Research, Innovate UK, Medical Research Council, and the British Heart Foundation. This trial was delivered by the Clinical Trial Evaluation Unit, now Bristol Trials Centre, a UK Clinical Research Collaboration registered clinical trials unit that is in receipt of National Institute of Health Research Clinical Trials Unit support funding. The views and opinions expressed in this report are those of the authors and do not necessarily reflect those of the National Institute of Health Research, National Health Service or the Department of Health and Social Care.

## Disclosures

None.

## Supporting information

Data S1
