# Supplementary material for: Low Frequency Ventilation During Cardiopulmonary Bypass to Protect Postoperative Lung Function in Cardiac Valvular Surgery: The PROTECTION Phase II Randomized Trial
Source: J Am Heart Assoc. 2024 Sep 30;13(19):e035011. doi: 10.1161/JAHA.124.035011 (PMC11681471; doi:10.1161/JAHA.124.035011)
Supplement: Supplementary file 1 — Data S1 [file JAH3-13-e035011-s001.pdf]

Supplemental Material

Data S1.

Supplemental Methods – additional information:

Protocol changes – timing of assessments

A month after the start of recruitment the protocol was revised; the timing of several assessments was changed:

- pulmonary function tests post-surgery was changed from *at discharge* to *between 5- and 7-days post-surgery*
- pulmonary gas exchange measurements were changed from *6 hours post CPB* to *4 hours post CPB*, and measurements gas post extubation, 12 hours post CPB, and before removal of arterial line were added
- respiratory system and lung mechanics measurements were changed from *on arrival in ICU, 2 and 6 hours post CPB* to *2 and 4 hours post end of CPB*
- Measurements timed to be taken *post CPB weaning* were changed to *post the end of CPB*

Protocol changes – trial interventions

As part of the same amendment submitted during the first month of recruitment aspects of the intervention were clarified to better reflect standard care:

- *Following a premed of temazepam 2030 mg* was replaced with *Following a premed as per standard care.*
- Anaesthesia will be maintained with *a combination of volatile anaesthetic agent.*
- *tidal volume of 8ml/Kg* was changed to *tidal volume of 68mL/Kg*'

- **…** normal control ventilation was changed from *to be reduced to 40% thereafter* to t*o be reduced to 50% thereafter*
- the standard cardiopulmonary bypass circuit priming solution was changed from *primed with 1000ml of Hartmann's solution, 500 mL of Gelofusine, 0.5 g/Kg mannitol, 7 mL of 10% calcium gluconate, and 6000 IU of heparin* to *primed with 1000 mL of Hartmann's solution, 500 mL of Gelofusine, 0.5 g/Kg mannitol, and 5000 IU of heparin*
- non pulsatile flow rates throughout bypass were changed from *2.4* L/m^2 *per minute* to *will be maintained according to routine standard care.*

Protocol changes – trial outcomes

As part of the amendment submitted during the first month of recruitment the secondary outcome of *oxygen in the blood measured by the intrapulmonary shunt fraction QS/QT***,** was changed to *oxygen in the blood measured by the Aa gradient***.**

A final amendment to the protocol occurred after recruitment was complete (July 2013), but before data lock and any analyses were undertaken. This amendment focussed on changes to trial outcomes, in particular the following outcomes were added:

- inflammatory mediators: sICAM-1, sVCAM-1, and S1P
- markers of direct lung epithelial/alveolar injury: sRAGE, SP-D, PAI-1, caspase-3, and quantitative fragmented mitochondrial DNA**.**

Trial outcomes not reported

The results of the laboratory analyses for thromboxane A and IL-6 are not reported as despite several attempts made by the senior lab scientists, including using different levels of dilution, it

was not possible to obtain readable levels for those 2 biomarkers only. This occurred before the protocol was amended to add markers to replace those that could not be included. Analyses of SP-D was not done due to insufficient funding for the laboratory consumables. Similarly, the markers of oxidative stress (malondialdehyde and conjugated dienes) and caspase-3 were not done as the analyses were not possible via the Laminex technology available to the research team. It also proved impossible to get reliable measurements for the quantitative fragmented mitochondrial DNA.

Anaesthesia and surgical methods – additional details

Surgery, anaesthesia and postoperative management was according to standardised protocols (32). Following a pre-medication, anaesthesia was induced with a combination of midazolam, propofol and fentanyl 5-10mcg/kg, and muscle relaxation was achieved with vecuronium or rocuronium. Anaesthesia was maintained with a combination of volatile anaesthetic agent, propofol (2-6mg/kg h) and fentanyl (5mcg/kg up to maximum of 20mcg/kg) as needed. After intubation, mechanical ventilation in both groups was the same up to institution of CPB and was guided by routine clinical practice entailing normal volume control ventilation (NV) at the following settings: tidal volume of 6-8 mL/kg, I/E ratio of 1:2, FiO_2 of 100% for 5 minutes to be reduced to 50% thereafter and ventilation rate of 12/minute aiming to obtain a favourable $PaCO_2$ (35.0 to 45.0 mmHg) and pH (7.35 to 7.45). For surgery, a standard CPB circuit was used, primed with 1000mL of Hartmann's solution, 500mL of Gelosine, 0.5 g/kg mannitol and 5000 IU of heparin. Non pulsatile flow rates throughout bypass were maintained according to routine standard care. Systemic temperature was between 30°C-34°C. Cardioplegic arrest was achieved and maintained with intermittent antegrade/retrograde cold blood cardioplegia (0-4°C).

Undertaking of mitral valve repair/replacement, aortic valve and coronary surgery was according to the surgeon's preference. All other aspects of the patient's care were standardised. Following surgery, patients were admitted to the CICU and managed by blind intensivists. Post-operative management in both groups was in accordance with standardised protocols. If the routine checks for body temperature, cardiovascular measurements, and blood loss were satisfactory, sedation was stopped and extubation was carried out per routine protocols. Antibiotic prophylaxis was given as per local protocol.

Statistical analysis – additional details

Continuous variates are summarised as mean and standard deviation (or median and interquartile range if the distribution is skewed. Category measures are reported as number and percentage. Missing data is included as footnotes to tables. Outcomes were compared using generalised linear (binary variables), Cox proportional hazards (time to event variables), or linear mixed (continuous variables measured at multiple time points) regression. Statistical model fit was assessed via standard methods (e.g. graphical plots) and if inadequate then transformations or alternative analysis methods were sought. Continuous outcomes analyzed on a logarithmic scale were transformed back to the original scale after analysis and results presented as geometric mean ratios (GMR). No sub-group analyses were planned.

Table S1. Withdrawals.

		Randomised to LFV (n=33)		Randomised to usual care (n=32)		Overall (N=65)	
		**n**	**%**	**n**	**%**	**n**	**%**
**Any withdrawal**		**1/33**	**1.5%**	**2/32**	**3.1%**	**3/65**	**4.6%**
Of those who withdrew:							
Time of withdrawal:	Intra-op	0/1	0.0%	2/2	100.0%	2/3	66.7%
	Post-op	1/1	100.0%	0/2	0.0%	1/3	33.3%
Decision taken by:	Patient	1/1	100.0%	0/2	0.0%	1/3	33.3%
	Clinician	0/1	0.0%	2/2	100.0%	2/3	66.7%
Reason for withdrawal:	Patient found to be ineligible	0/1	0.0%	2/2	100.0%	2/3	66.7%
	Patient felt it was all too much	1/1	100.0%	0/2	0.0%	1/3	33.3%

LFV=low frequency ventilation

Table S2. Additional baseline characteristics and preoperative medications

		Randomised to LFV (n=33)		Randomised to usual care (n=30)		Overall (n=63)	
		n	%	n	%	n	%
**BLOOD RESULTS**							
Haemoglobin	Median (IQR)	14	(12.9, 14.9)	13	(12.6, 14.0)	14	(12.7, 14.6)
Platelets	Median (IQR)	221	(178.0, 253.0)	208	(177.0, 240.0)	208	(177.0, 251.0)
Creatinine value	Median (IQR)	96	(87.0, 116.0)	86	(77.0, 100.0)	93	(82.0, 109.0)
WBC count	Median (IQR)	7	(6.7, 8.9)	7	(6.1, 9.7)	7	(6.6, 9.2)
CRP	CRP < 1	9/30	30.0%	7/28	25.0%	16/58	27.6%
	Median (IQR)	2	(2.0, 4.0)	2	(2.0, 3.0)	2	(2.0, 4.0)
**MEDICATIONS**							
Salbutamol		2/33	6.1%	2/30	6.7%	4/63	6.3%
Ipratropium bromide		0/33	0.0%	0/30	0.0%	0/63	0.0%
Beclamethasone		1/33	3.0%	2/30	6.7%	3/63	4.8%
Budesonide		0/33	0.0%	0/30	0.0%	0/63	0.0%
Fluticasone		1/33	3.0%	0/30	0.0%	1/63	1.6%
Tiotropium		0/33	0.0%	1/30	3.3%	1/63	1.6%
Salmeterol		1/33	3.0%	1/30	3.3%	2/63	3.2%
Formeterol		0/33	0.0%	0/30	0.0%	0/63	0.0%
Seretide		2/33	6.1%	0/30	0.0%	2/63	3.2%
Symbicort		0/33	0.0%	0/30	0.0%	0/63	0.0%
Fostair		0/33	0.0%	0/30	0.0%	0/63	0.0%
Aminophylline		0/33	0.0%	0/30	0.0%	0/63	0.0%
Monteleukast		0/33	0.0%	0/30	0.0%	0/63	0.0%
Azithromycin		0/33	0.0%	0/30	0.0%	0/63	0.0%
Other oral steroid		0/33	0.0%	0/30	0.0%	0/63	0.0%
Aspirin		11/33	33.3%	6/30	20.0%	17/63	27.0%
Clopidogrel		0/33	0.0%	0/30	0.0%	0/63	0.0%
Warfarin		9/33	27.3%	8/30	26.7%	17/63	27.0%

	Randomised to LFV (n=33)		Randomised to usual care (n=30)		Overall (n=63)	
	**n**	**%**	**n**	**%**	**n**	**%**
Heparin / clexane	1/33	3.0%	1/30	3.3%	2/63	3.2%
Beta blockers	13/33	39.4%	15/30	50.0%	28/63	44.4%
Calcium antagonists	4/33	12.1%	4/30	13.3%	8/63	12.7%
Oral nitrates	5/33	15.2%	0/30	0.0%	5/63	7.9%
IV GTN / nitrates	0/33	0.0%	0/30	0.0%	0/63	0.0%
Statins	14/33	42.4%	9/30	30.0%	23/63	36.5%
ACE inhibitors	10/33	30.3%	10/30	33.3%	20/63	31.7%
Angiotensin 2 blockers	3/33	9.1%	3/30	10.0%	6/63	9.5%
Diuretics	12/33	36.4%	16/30	53.3%	28/63	44.4%
Digoxin	6/33	18.2%	5/30	16.7%	11/63	17.5%
Oral anti-diabetics	3/33	9.1%	1/30	3.3%	4/63	6.3%
Insulin	1/33	3.0%	0/30	0.0%	1/63	1.6%
Anti-arrhythmic	4/33	12.1%	1/30	3.3%	5/63	7.9%

LFV=low frequency ventilation, WBC=White Blood Count; CRP=C-reactive protein; IV GTN=Intravenous infusion of Glyceryl Trinitrate; ACE=Angiotensin-converting enzyme; IQR=Interquartile Range; SD=Standard Deviation.

Table S3. Additional intra-operative and post-operative details

		Randomised to LFV (n=33)		Randomised to Usual care (n=30)		Overall (n=63)	
		n	%	n	%	n	%
**INTRA-OPERATIVE COLLOIDS**							
Hydroxyethyl starch (HES/ HAES)		2/33	6.1%	2/30	6.7%	4/63	6.3%
Gelofusine		19/33	57.6%	8/30	26.7%	27/63	42.9%
**CPB PRIME COMPOSITION**							
1000ml Hartmanns		32/33	97.0%	30/30	100.0%	62/63	98.4%
500ml Gelofusine		31/33	93.9%	30/30	100.0%	61/63	96.8%
5000 units Heparin		32/33	97.0%	30/30	100.0%	62/63	98.4%
20% Mannitol 0.5g/Kg		32/33	97.0%	30/30	100.0%	62/63	98.4%
**EXTRA FLUID ADMINISTERED INTO CPB DURING SURGERY**							
Gelofusine		9/33	27.3%	8/30	26.7%	17/63	27.0%
Hartmanns		5/33	15.2%	3/30	10.0%	8/63	12.7%
Sodium Bicarbonate		4/33	12.1%	4/30	13.3%	8/63	12.7%
**RETURN FROM THEATRE**							
Haematocrit (%)	Median (IQR)	30	(26.9, 31.1)	29	(26.0, 30.4)	29	(26.1, 30.8)
Lactate (mmol/l)	Median (IQR)	1	(1.1, 2.0)	1	(1.0, 1.4)	1	(1.1, 1.7)
Temperature	Median (IQR)	36	(35.8, 36.4)	36	(35.5, 36.3)	36	(35.7, 36.4)
**BLOOD RESULTS IN FIRST 24 HOURS**							
Lowest haemoglobin (g/dL)	Median (IQR)	9	(7.6, 9.6)	8	(7.4, 8.7)	8	(7.6, 9.3)
Lowest haematocrit (%)	Median (IQR)	26	(23.5, 29.2)	25	(23.0, 27.0)	25	(23.4, 28.6)
Lowest MABP (mm Hg)	Median (IQR)	62	(56.0, 64.0)	58	(55.0, 65.0)	60	(55.0, 65.0)
Highest lactate (mmol/l)	Median (IQR)	2	(1.8, 2.8)	3	(1.9, 3.4)	3	(1.9, 3.4)
Fluid balance at 12 hours (mL)		552	(175, 817)	640	(214, 1160)	569	(195, 1019)
Total chest tube drainage (mL)		625	(400, 900)	725	(425, 900)	675	(425, 900)

		Randomised to LFV (n=33)		Randomised to Usual care (n=30)		Overall (n=63)	
		**n**	**%**	**n**	**%**	**n**	**%**
Post-operative insulin infusion		22/33	66.7%	18/30	60.0%	40/63	63.5%
**BLOOD PRODUCT USE POST-OPERATIVELY**							
RBC transfusion		11/33	33.3%	15/30	50%	26/63	41.3%
Units transfused	Median (IQR)	3	(2, 5)	2	(1, 4)	3	(2, 4)
FFP transfusion		4/33	12.1%	2/30	6.7%	6/63	9.5%
Units transfused	Median (IQR)	3	(1.5, 4.5)	2.5	(2, 3)	2.5	(2, 4)
Platelet transfusion		7/33	21.2%	2/30	6.7%	9/63	14.3%
Units transfused	Median (IQR)	1	(1, 2)	1	(1, 1)	1	(1, 2)
Cryoprecipitate transfusion		2/33	6.1%	2/30	6.7%	4/63	6.4%
Units transfused	Median (IQR)	2	(2, 2)	2	(2, 2)	2	(2, 2)
**BLOOD PRODUCT USE FOLLOWING RE-OPERATION**							
RBC transfusion		4/5		1/2		5/7	
Units transfused	Median (IQR)	2	(1, 4)	3		2.5	(1, 4)
FFP transfusion		4/5		1/2		5/7	
Units transfused	Median (IQR)	2	(2, 3)	5		2	(2, 4)
Platelet transfusion		3/5		1/2			
Units transfused	Median (IQR)	1	(1, 1)	2		1	(1, 1.5)
Cryoprecipitate transfusion		1/5		1/2		2/7	
Units transfused	Median (IQR)	2		2		2	
Post-operative inotropes (excl. Noradrenaline)		10/33	30.3%	10/30	33.3%	20/63	31.7%
Post-operative noradrenalin		17/33	51.5%	18/30	60.0%	35/63	55.6%
**INTUBUTION AND CARDIAC INTENSIVE CARE UNIT STAY AND DISCHARGE DESTINATION**							
Duration of intubation (hours)	Median (IQR)	6.75	(4.25, 9.75)	7.50	(4.92, 13.30)	7.00	(4.25, 12.63)

		Randomised to LFV (n=33)		Randomised to Usual care (n=30)		Overall (n=63)	
		**n**	**%**	**n**	**%**	**n**	**%**
Duration of cardiac intensive care unit stay (hours)	Median (IQR)	95.8	(53.0, 119.6)	74.2	(55.7, 119.6)	91.0	(55.2, 119.6)
Discharge destination from cardiac unit	Home	32/33	97.0%	28/30	93.3%	60/63	95.2%
	Other hospital	1/33	3.0%	2/30	6.7%	3/63	4.8%

LFV=low frequency ventilation, RBC=Red Blood Cell; FFP=Fresh Frozen Plasma; PLT=Platelet; MABP=Mean arterial blood pressure

Note: *No participant received activated factor VII*

Table S4. Primary outcome: inflammatory markers and tissue injury mediators

	Timepoint	Randomised to LFV (n=33)		Randomised to Usual care (n=30)		GMR*	
		Median	IQR	Median	IQR	(95% CI)	p-value
TNFα[1]	Pre-sternotomy	2.92	1.99, 3.69	2.81	2.16, 4.91		
	10 mins post CPB	6.78	3.90, 14.62	8.96	5.89, 13.87		
	2 hours post CPB	11.75	7.72, 22.19	14.24	9.34, 20.24		
	6 hours post CPB	6.58	4.34, 10.32	7.78	5.10, 13.32		
	12 hours post CPB	4.15	2.66, 6.49	3.70	3.14, 6.50		
	24 hours post CPB	3.94	2.39, 5.65	4.25	2.71, 6.09		
	Treatment*Time interaction						0.89
	Overall					0.91 (0.75, 1.09)	0.27
IL-1β[2]	Pre-sternotomy	0.55	0.23, 0.76	0.49	0.22, 0.87		
	10 mins post CPB	0.16	0.06, 0.36	0.18	0.10, 0.45		
	2 hours post CPB	0.23	0.08, 0.39	0.14	0.10, 0.36		
	6 hours post CPB	0.32	0.17, 0.49	0.32	0.20, 0.45		
	12 hours post CPB	0.37	0.22, 0.66	0.47	0.28, 0.72		
	24 hours post CPB	0.40	0.23, 0.54	0.48	0.20, 0.76		
	Treatment*Time interaction						0.82
	Overall					0.95 (0.66, 1.37)	0.75
IL-10[3]	Pre-sternotomy	8.23	4.30, 13.81	10.99	6.76, 16.07		
	10 mins post CPB	2634.28	991.04, 5056.87	1608.11	1054.08, 3654.62		
	2 hours post CPB	534.18	158.03, 909.53	276.20	127.86, 565.30		
	6 hours post CPB	61.02	46.78, 153.04	79.98	47.00, 117.96		
	12 hours post CPB	72.71	32.72, 183.30	70.96	43.62, 152.81		
	24 hours post CPB	44.70	22.83, 91.95	40.55	33.60, 73.18		
	Treatment*Time interaction						0.59
	Overall					1.22 (0.89, 1.68)	0.189
sICAM-1	Pre-sternotomy	2781	(2328, 3494)	3054	(2597, 4106)		
	10 mins post CPB	1955	(1678, 2563)	2349	(1671, 2982)		
	2 hours post CPB	2199	(1714, 2909)	2444	(1680, 3166)		

	Timepoint	Randomised to LFV (n=33)		Randomised to Usual care (n=30)		GMR*	
		Median	IQR	Median	IQR	(95% CI)	p-value
	6 hours post CPB	2931	(2380, 3303)	3095	(2169, 4343)		
	12 hours post CPB	2941	(2374, 3634)	3433	(2627, 4478)		
	24 hours post CPB	3104	(2640, 3802)	3410	(2663, 5015)		
	Treatment*Time interaction						0.29
	Overall					1.03 (0.92, 1.16)	0.60
sVCAM-1	Pre-sternotomy	10657	(8876, 14181)	11572	(9929, 13691)		
	10 mins post CPB	11009	(7301, 13081)	10219	(9299, 11800)		
	2 hours post CPB	11736	(8842, 15175)	11618	(9787, 14203)		
	6 hours post CPB	15053	(12550, 17595)	14349	(12162, 16305)		
	12 hours post CPB	15452	(11586, 19615)	16015	(13609, 17873)		
	24 hours post CPB	15245	(11410, 19371)	15673	(13225, 19385)		
	Treatment*Time interaction						0.19
	Overall					0.99 (0.89, 1.11)	0.92
S1P	Pre-sternotomy	0.35	0.27, 0.55	0.32	0.21, 0.49		
	10 mins post CPB	0.22	0.17, 0.33	0.21	0.14, 0.34		
	2 hours post CPB	0.25	0.17, 0.37	0.23	0.17, 0.32		
	6 hours post CPB	0.28	0.21, 0.39	0.24	0.16, 0.36		
	12 hours post CPB	0.23	0.15, 0.44	0.28	0.20, 0.34		
	24 hours post CPB	0.21	0.16, 0.42	0.26	0.16, 0.37		
	Treatment*Time interaction						0.40
	Overall					0.98 (0.87, 1.12)	0.786
sRAGE	Post-induction	104.5	54.48, 138.6	119.3	69.61, 363.9		
	10 minutes post end of CPB	8948	483.6, 15698	876.1	181.7, 11289	3.05 (1.13, 8.24)	0.026
	2 hrs post CPB	200.7	124.9, 393.7	241.1	76.8, 597.0	1.07 (0.64, 1.79)	0.797
	6 hrs post CPB	92.27	58.15, 126.5	101.8	32.42, 256.7	0.84 (0.55, 1.27)	0.388
	12 hrs post CPB	69.37	37.98, 102.7	100.6	43.30, 172.8	0.67 (0.42, 1.07)	0.083
	24 hrs post CPB	56.25	35.04, 77.34	89.00	44.21, 143.8	0.62 (0.45, 0.85)	0.003

	Timepoint	Randomised to LFV (n=33)		Randomised to Usual care (n=30)		GMR* (95% CI)	p-value
		Median	IQR	Median	IQR		
	Treatment*Time interaction						0.025
tPAI-1	Post-induction	957.2	735.8, 1230	696.4	475.8, 1391.27		
	10 minutes post end of CPB	1160.1	882.2, 1492	1136.4	686.09, 1420.99		
	2 hrs post CPB	2998.0	1323, 5176	1751.1	907.11, 2705.84		
	6 hrs post CPB	4511.8	3290, 6906	7034.8	4155.53, 9629.61		
	12 hrs post CPB	1992.3	1121, 3567	2790.6	1567.75, 4903.65		
	24 hrs post CPB	1809.4	1042, 2368	1636.7	940.20, 3194.55		
	Treatment*Time interaction						0.40
	Overall					MD = 81.5 (-431.8, 594.8)	0.72

LFV=low frequency ventilation , TNFα=tumour necrosis factor alpha; IL1 β=interleukin 1 beta, IL-10=interleukin-10), sICAM-1=soluble intercellular adhesion molecules-1 sVCAM-1= soluble vascular cell adhesion molecules-1, S1P sphingosine-1-phosphate. sRAGE=soluble receptor for advance glycation end products, tPAI-1=plasminogen activator inhibitor-1, LFV=low frequency ventilation, IQR=interquartile range, GMR=Geometric mean ratio, MD=mean difference, CI=confidence interval

Note: *treatment effect estimates are given either for each time point or overall, depending on if a treatment*time interaction term is found to be required in the model.*

Missing data (LFV, UC): [1]1(1, 0), [2] 18(13, 5), [3]3(2, 1)

Table S5. Lung complications and 6-min walking distance before discharge

		Randomised to LFV (n=33)		Randomised to usual care (n=30)		Effect[1] (95% CI)	p-value
		**n**	**%**	**n**	**%**		
Composite endpoint of lung-related complications		5/33	15.2%	4/30	13.3%	RR = 1.24<br>(0.39, 3.91)<br>OR = 1.32<br>(0.30, 5.91)	p=0.71<br><br><br>p=0.71
Bacteriologically proven chest infection		3/33	9.1%	0/30	0.0%		
Prolonged ventilation (>24 hours)		3/33	9.1%	2/30	6.7%		
Requirement of CPAP		1/33	3.0%	2/30	6.7%		
Need for re-intubation		1/33	3.0%	0/30	0.0%		
Tracheostomy		0/33	0.0%	0/30	0.0%		
ARDS		0/33	0.0%	0/30	0.0%		
6MWT*	Mean (SD)	256	99.7	195	79.5	MD = 63.2<br>(12.9, 113.6)	0.012
Number of patients who reached 'fitness for discharge' prior to discharge		11/33	33.3%	10/30	33.3%		
Time until fit for discharge[1]	Median (IQR	6	(5, 7)	8.5	(5, 9)	HR = 1.31<br>(0.55, 3.16)	0.54
Time to hospital discharge	Median (IQR)	7	(6, 10)	8	(7, 12)	HR = 0.98<br>(0.59, 1.65)	0.95

** in patients who reached fitness for discharge*

LFV=low frequency ventilation, ARDS=acute respiratory distress syndrome, CPAP=continuous positive airway pressure, 6MWT+ 6 min walking test, SD=standard deviation, IQR=interquartile range, RR=risk ratio, OR=odds ratio. MD=mean difference, HR=hazard ratio, CI=confidence interval

Missing data (LFV, UC) [1]12 patients (10, 2).

Table S6. Post-operative complications and SAE at 6 to 8 weeks after discharge

Number of participants	Randomised to LFV (n=33)				Randomised to usual care (n=30)			
	Adverse Event		Serious Adverse Event		Adverse Event		Serious Adverse Event	
	n	%	n	%	n	%	n	%
At least one in-hospital event	33/33	100.0%	10/32	31.3%	28/30	93.3%	11/29	37.9%
At least one SAE post-discharge			11/30	36.7%			8/28	28.6%
At least one SAE at any time			17/30	56.7%			16/27	59.3%
**In-hospital events**								
Cardiac arrest	1/33	3.0%	1/33	3.0%	1/30	3.3%	1/30	3.3%
Resuscitation attempted	1/1	100.0%			1/1	100.0%		
Resuscitation successful	1/1	100.0%			1/1	100.0%		
SVT/AF requiring treatment	18/33	54.5%	3/33*	9.1%	13/30	43.3%	3/30	10.0%
VF/VT requiring intervention	1/33	3.0%	0/33	0.0%	1/30	3.3%	1/30	3.3%
New pacing	22/33	66.7%	2/33	6.1%	16/30	53.3%	4/30	13.3%
Single pacing	5/22	22.7%			7/16	43.8%		
Double pacing	17/22	77.3%			9/16	56.3%		
Temporary pacing become permanent	2/22	9.1%			1/16	6.3%		
Reoperation	5/33	15.2%	2/32	6.3%	2/30	6.7%	1/29	3.4%
Chest re-opened?	5/5	100.0%			2/2	100.0%		
Tamponade	3/5	60.0%			0/2	0.0%		
Bleeding	2/5	40.0%			1/2	50.0%		
Cardiac arrest	0/5	0.0%			1/2	50.0%		
Mediastinitis	1/5	20.0%			0/2	0.0%		
Any inotropes used	19/33	57.6%	3/33	9.1%	17/30	56.7%	6/30	20.0%

Number of participants	Randomised to LFV (n=33)				Randomised to usual care (n=30)			
	Adverse Event		Serious Adverse Event		Adverse Event		Serious Adverse Event	
	**n**	**%**	**n**	**%**	**n**	**%**	**n**	**%**
IABP inserted	0/33	0.0%	0/33	0.0%	1/30	3.3%	0/30	0.0%
Pulmonary artery catheter inserted	0/33	0.0%	0/33	0.0%	1/30	3.3%	0/30	0.0%
Vasodilator used	7/33	21.2%	1/33	3.0%	10/30	33.3%	1/30	3.3%
Low cardiac output	4/33	12.1%	3/33	9.1%	3/30	10.0%	3/30	10.0%
Re-intubation and ventilation required	1/33**	3.0%	1/33	3.0%	0/30	0.0%	0/30	0.0%
Mask CPAP	1/33	3.0%	0/33	0.0%	2/30	6.7%	1/30	3.3%
Prolonged ventilation >24Hrs	3/33	9.1%	2/33	6.1%	2/30	6.7%	2/30	6.7%
Pneumothorax or effusion requiring drainage	1/33	3.0%	1/33	3.0%	1/30	3.3%	0/30	0.0%
TIA	1/33	3.0%	0/33	0.0%	0/30	0.0%	0/30	0.0%
Excess bleeding, not requiring re-operation	1/33	3.0%	0/33	0.0%	1/30	3.3%	1/30	3.3%
Wound dehiscence requiring rewiring or treatment	1/33	3.0%	1/33	3.0%	0/30	0.0%	0/30	0.0%
Infective complications	9/33	27.3%	4/32	12.5%	7/30	23.3%	0/26	0.0%
Sputum	3/9	33.3%			4/7***	57.1%		
Surgical wound	3/9****	33.3%			2/7	28.6%		
Urine	1/9	11.1%			1/7	14.3%		
Bowel infection	1/9	11.1%			0/7	0.0%		
Other	2/9	22.2%			0/7	0.0%		
Unknown	0/9	0.0%			1/7	14.3%		

Number of participants	Randomised to LFV (n=33)				Randomised to usual care (n=30)			
	Adverse Event		Serious Adverse Event		Adverse Event		Serious Adverse Event	
	**n**	**%**	**n**	**%**	**n**	**%**	**n**	**%**
Bacteriologically confirmed?	6/9	66.7%			0/7	0.0%		
Hypotension			0/33	0.0%			1/30	3.3%
Confusion			0/33	0.0%			1/30	3.3%
Diarrhoea			1/33	3.0%			0/30	0.0%
Hyponatraemia (low sodium levels)			1/33	3.0%			0/30	0.0%
Loss of vision			1/33	3.0%			0/30	0.0%
**Post-discharge events**								
SVT/AF			2/31	6.5%			0/29	0.0%
Pacing (double)			0/31	0.0%			1/29	3.4%
Temporary pacing became permanent							1/1	100.0%
Low heart rate			0/33	0.0%			1/30	3.3%
Pneumothorax or effusion			1/31	3.2%			3/29	10.3%
Persistent pleural effusions			0/33	0.0%			1/30	3.3%
Fluid in pericardium			1/33	3.0%			0/30	0.0%
Infective complications			8/30	26.7%			5/29	17.2%
Sputum			1/7	14.3%			0/5	0.0%
Surgical wound			4/7	57.1%			2/5	40.0%
Urine			0/7	0.0%			1/5	20.0%
Other			2/7	28.6%			1/5	20.0%
Unknown			1/8	12.5%			0/5	0.0%

Number of participants	Randomised to LFV (n=33)				Randomised to usual care (n=30)			
	Adverse Event		Serious Adverse Event		Adverse Event		Serious Adverse Event	
	n	%	n	%	n	%	n	%
Bacteriologically confirmed?			2/8	25.0%			1/5	20.0%
Toe infection			1/33	3.0%			0/30	0.0%
Rigors and malaise			0/33	0.0%			1/30	3.3%
Dizziness/double vision			1/33	3.0%			0/30	0.0%

SAE=serious adverse event, LFV=low frequency ventilation, SVT/AF= supraventricular tachycardia/atrial fibrillation; VF/VT=ventricular fibrillation/ventricular tachycardia; IABP=intra-aortic balloon pump; CPAP=continuous positive airway pressure; TIA=transient ischemic attack. ***Notes****: there were no cases of myocardial infarction, tracheostomy, acute respiratory distress syndrome renal failure requiring dialysis, gastrointestinal complications, deep vein thrombosis, pulmonary embolus, or infections in the blood*

** One participant with SVT/AF SAE reported twice in-hospital*

*** One participant with reintubation recorded twice*

**** One participant with three incidences of sputum infection reported*

***** One participant with four incidences of wound infection; one patient with two incidences of wound infection*

Table S7. Serious adverse events not listed in the study protocol

		Randomised to LFV (n=33)		Randomised to usual care (n=30)	
		**n**	**%**	**n**	**%**
**Number of patients experiencing an unexpected SAE**		5	15.2%	5	16.6%
**Number of unexpected SAES**		**6**		**5**	
Hypotension		0		1	
Low heart rate		0		1	
Persistent pleural effusions		0		1	
Fluid in pericardium		1		0	
Confusion		0		1	
Hyponatraemia (low sodium levels)		1		0	
Diarrhoea		1		0	
Toe infection		1		0	
Loss of vision		1		0	
Rigors and malaise		0		1	
Dizziness/double vision		1		0	
**Timing of events**	Post-surgery but pre-discharge	3	9.1%	2	6.7%
	Post-discharge	3	9.1%	3	10.0%
**Maximum intensity**	Moderate	4	12.1%	1	3.3%
	Severe	2	6.1%	4	13.3%
**Reason event classified as SAE**	Resulted in death	0	0.0%	0	0.0%
	Is/was life threatening	0	0.0%	2	6.7%
	Resulted in persistent or significant disability/incapacity	3	9.1%	3	10.0%
	Prolonged ongoing hospitalisation/ caused hospitalisation	4	12.1%	4	13.3%

**Relatedness to *study***	Not related	3	9.1%	3	10.0%
treatment	Unlikely to be related	3	9.1%	2	6.7%

LFV=low frequency ventilation, SAE=serious adverse event,

***Note**: an unexpected SAE is an event that was not listed as expected in the study protocol, these events are also included in Table 3 of the main manuscript*

Figure S1. Primary biochemical outcomes.

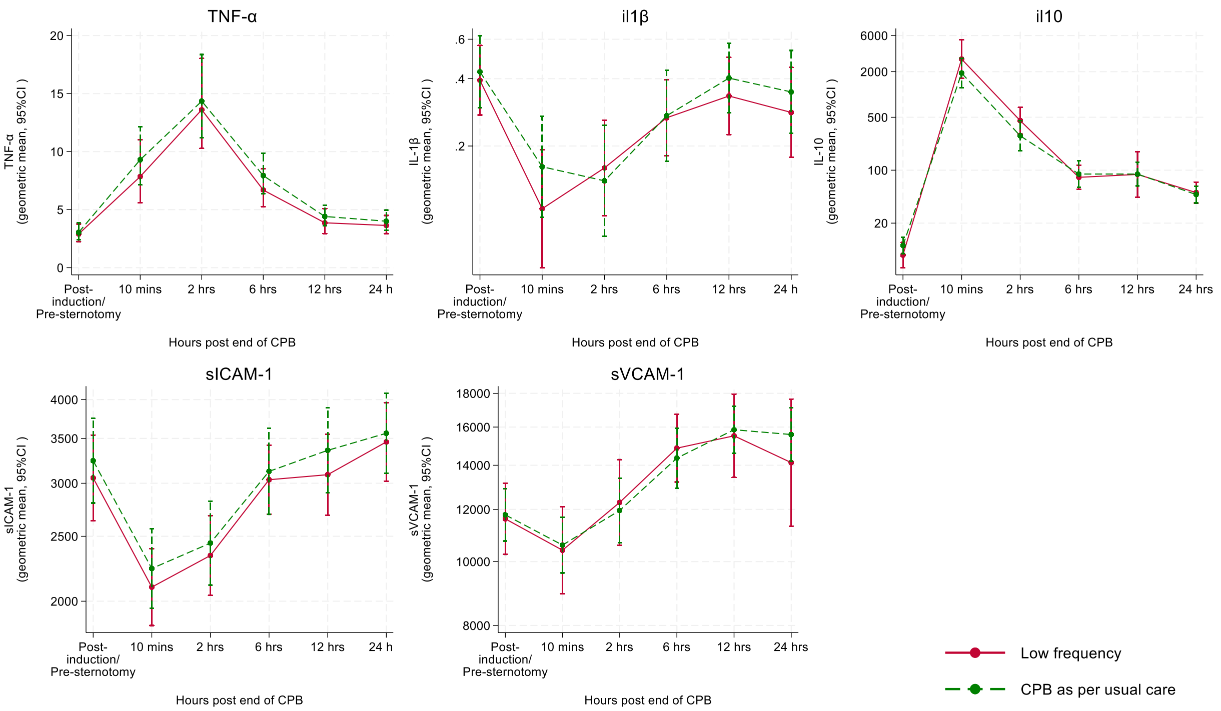

Figure shows the geometric mean and 95% confidence interval for each outcome

Figure S2. Primary biochemical outcomes.

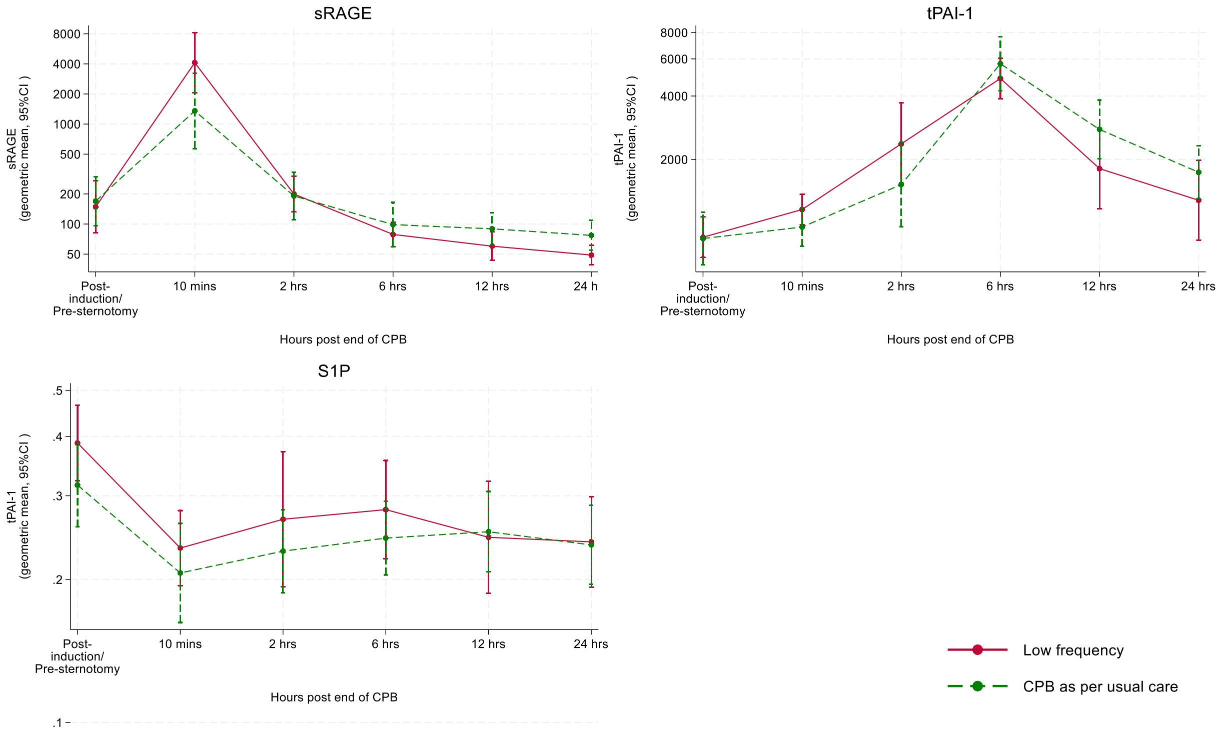

Figure S3. Pulmonary Function Tests.

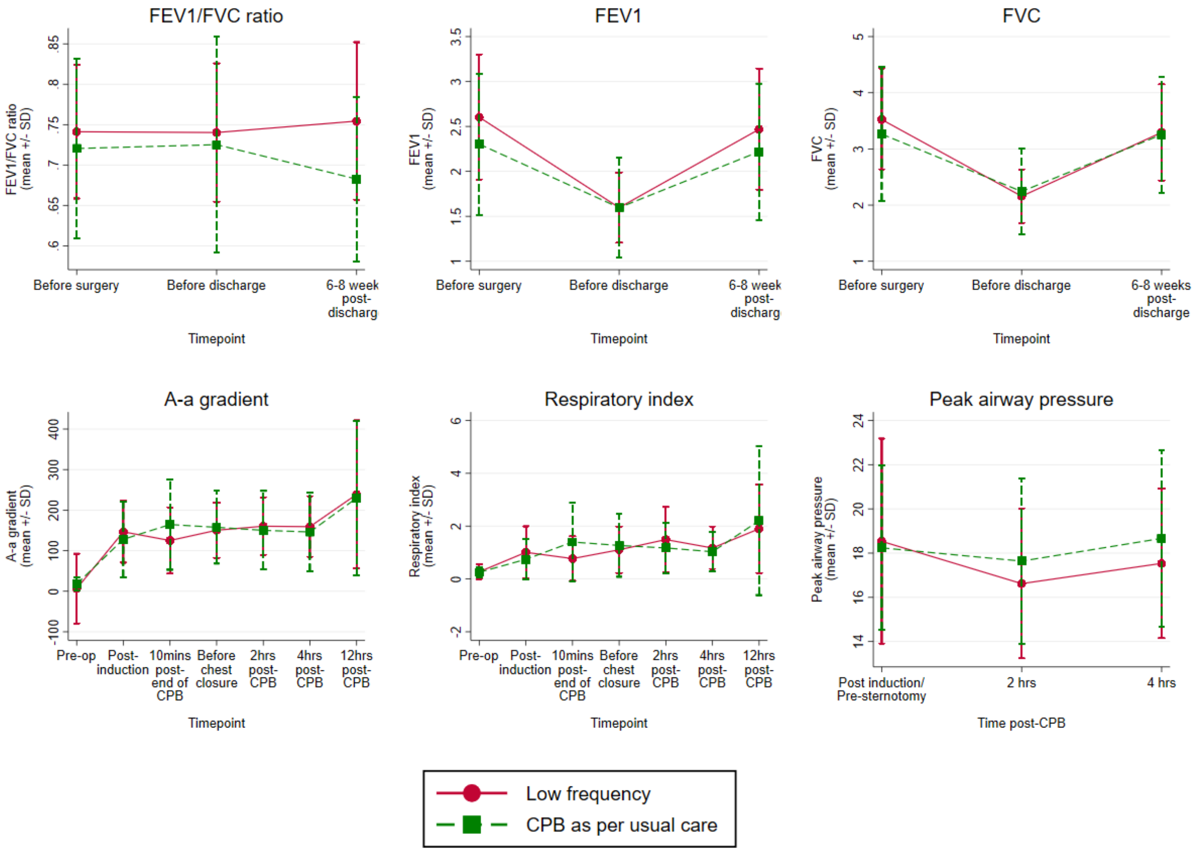

Figure shows the mean +/- standard deviation for each outcome
